# (2,4-Difluoro­phen­yl)[1-(1*H*-1,2,4-triazol-1-yl)cyclo­prop­yl]methanone

**DOI:** 10.1107/S1600536811040037

**Published:** 2011-10-12

**Authors:** Chunli Wu, Wei Lei, Huiyan Ma, Jiabin Qiao, Aixing Li

**Affiliations:** a School of Pharmaceutical Sciences, Zhengzhou Univresity, Zhengzhou 450001, People’s Republic of China

## Abstract

The asymmetric unit of the title compound, C_12_H_9_F_2_N_3_O, contains two independent mol­ecules (*A* and *B*) in which the benzene and cyclo­propane rings form dihedral angles of 33.0 (1) and 29.7 (1)°, respectively. In the crystal, weak inter­molecular C—H⋯O hydrogen bonds link alternating *A* and *B* mol­ecules into chains along [010].

## Related literature

For applications of triazole derivatives, see: Che & Zhang (2009 ▶); Lieven & Leo (2005 ▶). For related structures, see: Tarun *et al.* (1998 ▶).
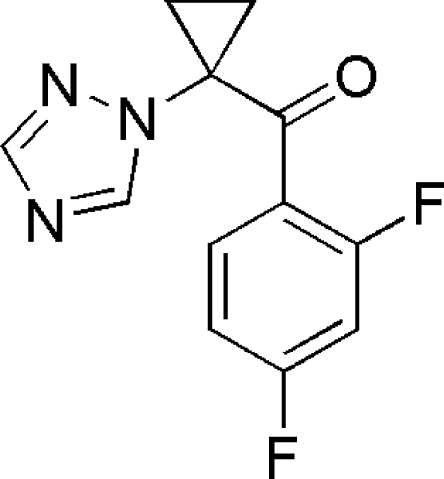


## Experimental

### 

#### Crystal data

C_12_H_9_F_2_N_3_O
*M* *_r_* = 249.22
Triclinic, 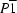

*a* = 9.6067 (11) Å
*b* = 11.4840 (13) Å
*c* = 11.9127 (14) Å
α = 73.652 (1)°
β = 84.202 (2)°
γ = 69.260 (1)°
*V* = 1179.4 (2) Å^3^
*Z* = 4
Mo *K*α radiation
μ = 0.12 mm^−1^
*T* = 298 K
0.49 × 0.40 × 0.38 mm

#### Data collection

Rigaku R-AXIS CCD detector diffractometer
Absorption correction: multi-scan (*ABSCOR*; Higashi, 1995 ▶) *T*_min_ = 0.983, *T*_max_ = 0.986
5900 measured reflections
4080 independent reflections
2816 reflections with *I* > 2σ(*I*)
*R*_int_ = 0.017

#### Refinement

*R*[*F* ^2^ > 2σ(*F* ^2^)] = 0.038
*wR*(*F* ^2^) = 0.111
*S* = 1.02
4080 reflections
326 parameters
H-atom parameters constrained
Δρ_max_ = 0.14 e Å^−3^
Δρ_min_ = −0.16 e Å^−3^

### 

Data collection: *R-AXIS II Software* (Rigaku, 1997 ▶); cell refinement: *R-AXIS II Software*; data reduction: *R-AXIS II Software*; program(s) used to solve structure: *SHELXS97* (Sheldrick, 2008 ▶); program(s) used to refine structure: *SHELXL97* (Sheldrick, 2008 ▶); molecular graphics: *TEXSAN* (Molecular Structure Corporation, 1992 ▶); software used to prepare material for publication: *SHELXL97*.

## Supplementary Material

Crystal structure: contains datablock(s) I, global. DOI: 10.1107/S1600536811040037/cv5156sup1.cif

Structure factors: contains datablock(s) I. DOI: 10.1107/S1600536811040037/cv5156Isup2.hkl

Supplementary material file. DOI: 10.1107/S1600536811040037/cv5156Isup3.cml

Additional supplementary materials:  crystallographic information; 3D view; checkCIF report

## Figures and Tables

**Table 1:** Hydrogen-bond geometry (Å, °)

*D*—H⋯*A*	*D*—H	H⋯*A*	*D*⋯*A*	*D*—H⋯*A*
C12—H12*A*⋯O2^i^	0.93	2.35	3.268 (2)	171
C24—H24*A*⋯O1^ii^	0.93	2.37	3.265 (3)	161

Symmetry codes: (i) 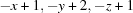; (ii) 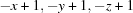.

**Fig. 1.**
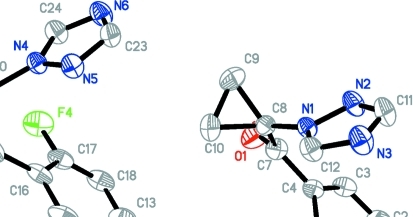
Two independent molecules of (I) showing the atomic numbering and 30% probability displacement ellipsoids.

## supplementary crystallographic information

### Comment

The triazole derivatives exhibit various  antifungal activities (Che & Zhang, 2009; Lieven &Leo, 2005). As our contribution to the research of triazole compounds, herewith we report the crystal structure of the title compound (I).

The asymmetric unit of (I) contains two independent molecules, A and B, respectively (Fig. 1). The benzene and cyclopropane rings form a dihedral angle of 33.0 (1)° in A and 29.7 (1)° in B. All bond lengths and angles in (I) are normal and comparable with those found in related compounds (Tarun *et al.*, 1998). In the crystal structure, weak intermolecular C—H···O hydrogen bonds (Table 1) link alternating A and B molecules into chains in [010].

### Experimental

To a suspension of 2.27 g (10 mmol) of 2-(1*H*-1,2,4- triazol)-2',4'-difluoroacetophenone and 1.68 g (30 mmol) of KOH in 60 ml of stirred acetone, was cautiously added 2.6 ml (30 mmol) of 1,2-dibromoethane. After the mixture was stirred at room temperature for 6 hr, the mixture was filtered,and then solvent was evaporated. The crystalline product was separated by chromatographic column(petroleum ether:acetone 6:1) with yield 30%. Crystals suitable for X-ray analysis were grown by slow evaporation from acetone at room temperature for two weeks. M.p. 89–90 °C. Spectroscopic analysis: ^1^H NMR (400 MHz, CDCl_3_) σ: 8.21(s, 1H), 7.80(s, 1H), 7.40(1*H*, dd, *J*=14.5 8.2 Hz), 6.87(m, 1H), 6.73(m, 1H), 2.13(1*H*, q, *J*=5.0 Hz), 1.80(1*H*, q, *J*=5.0 Hz). ^13^C NMR (100 MHz, CDCl_3_) σ: 196.47, 165.45, 165.33, 162.95, 162.83, 160.62, 160.50, 158.12, 157.99, 152.33, 146.44, 130.94, 130.89, 130.83, 130.79, 122.71, 122.67, 122.55, 122.51, 112.48, 112.44, 112.26, 112.23, 105.25, 104.99, 104.73, 49.23, 19.04.

### Refinement

All H atoms were placed geometrically and treated as riding on their parent atoms with C—H are 0.96 Å (methylene) or 0.93 Å (aromatic), and *U*_iso_(H) =1.2*U*_eq_(C).

### Crystal data

**Table:**

C_12_H_9_F_2_N_3_O	*F*(000) = 512
*M**_r_* = 249.22	*D*_x_ = 1.404 Mg m^−^^3^
Triclinic, *P*1	Melting point: 362.15 K
*a* = 9.6067 (11) Å	Mo *K*α radiation, λ = 0.71073 Å
*b* = 11.4840 (13) Å	Cell parameters from 2449 reflections
*c* = 11.9127 (14) Å	θ = 4.9–52.5°
α = 73.652 (1)°	µ = 0.12 mm^−^^1^
β = 84.202 (2)°	*T* = 298 K
γ = 69.260 (1)°	Block, colourless
*V* = 1179.4 (2) Å^3^	0.49 × 0.40 × 0.38 mm
*Z* = 4	

### Data collection

**Table:**

Rigaku R-AXIS CCD detector diffractometer	4080 independent reflections
Radiation source: fine-focus sealed tube	2816 reflections with *I* > 2σ(*I*)
graphite	*R*_int_ = 0.017
φ and ω scans	θ_max_ = 25.0°, θ_min_ = 2.4°
Absorption correction: multi-scan (*ABSCOR*; Higashi, 1995)	*h* = −10→11
*T*_min_ = 0.983, *T*_max_ = 0.986	*k* = −13→12
5900 measured reflections	*l* = −13→14

### Refinement

**Table:**

Refinement on *F*^2^	Secondary atom site location: difference Fourier map
Least-squares matrix: full	Hydrogen site location: inferred from neighbouring sites
*R*[*F*^2^ > 2σ(*F*^2^)] = 0.038	H-atom parameters constrained
*wR*(*F*^2^) = 0.111	*w* = 1/[σ^2^(*F*_o_^2^) + (0.0469*P*)^2^ + 0.2296*P*] where *P* = (*F*_o_^2^ + 2*F*_c_^2^)/3
*S* = 1.02	(Δ/σ)_max_ < 0.001
4080 reflections	Δρ_max_ = 0.14 e Å^−^^3^
326 parameters	Δρ_min_ = −0.16 e Å^−^^3^
0 restraints	Extinction correction: *SHELXL97* (Sheldrick, 2008), Fc^*^=kFc[1+0.001xFc^2^λ^3^/sin(2θ)]^-1/4^
Primary atom site location: structure-invariant direct methods	Extinction coefficient: 0.0200 (19)

### Special details

**Table:**

Geometry. All e.s.d.'s (except the e.s.d. in the dihedral angle between two l.s. planes) are estimated using the full covariance matrix. The cell e.s.d.'s are taken into account individually in the estimation of e.s.d.'s in distances, angles and torsion angles; correlations between e.s.d.'s in cell parameters are only used when they are defined by crystal symmetry. An approximate (isotropic) treatment of cell e.s.d.'s is used for estimating e.s.d.'s involving l.s. planes.
Refinement. Refinement of *F*^2^ against ALL reflections. The weighted *R*-factor *wR* and goodness of fit *S* are based on *F*^2^, conventional *R*-factors *R* are based on *F*, with *F* set to zero for negative *F*^2^. The threshold expression of *F*^2^ > σ(*F*^2^) is used only for calculating *R*-factors(gt) *etc*. and is not relevant to the choice of reflections for refinement. *R*-factors based on *F*^2^ are statistically about twice as large as those based on *F*, and *R*- factors based on ALL data will be even larger.

### Fractional atomic coordinates and isotropic or equivalent isotropic displacement parameters (Å^2^)

**Table:**

	*x*	*y*	*z*	*U*_iso_*/*U*_eq_	
F1	1.40947 (15)	0.64219 (16)	0.00685 (15)	0.1017 (5)	
F2	0.96387 (13)	0.81942 (11)	0.18986 (12)	0.0717 (4)	
F3	0.93617 (18)	0.62666 (18)	0.51796 (17)	0.1195 (6)	
F4	0.49568 (16)	0.58407 (11)	0.69708 (13)	0.0857 (4)	
C1	1.2783 (2)	0.6334 (2)	0.0552 (2)	0.0647 (6)	
C2	1.2443 (2)	0.5265 (2)	0.0588 (2)	0.0668 (6)	
H2A	1.3102	0.4597	0.0302	0.080*	
C3	1.1099 (2)	0.52091 (19)	0.10591 (18)	0.0584 (5)	
H3A	1.0851	0.4485	0.1102	0.070*	
C4	1.0094 (2)	0.62149 (17)	0.14757 (16)	0.0478 (5)	
C5	1.0544 (2)	0.72398 (18)	0.14347 (17)	0.0512 (5)	
C6	1.1876 (2)	0.7330 (2)	0.09827 (18)	0.0591 (6)	
H6A	1.2151	0.8033	0.0969	0.071*	
C7	0.8640 (2)	0.61067 (19)	0.19488 (18)	0.0556 (5)	
C8	0.7238 (2)	0.72473 (17)	0.16397 (16)	0.0463 (5)	
C9	0.5788 (2)	0.7042 (2)	0.1994 (2)	0.0678 (6)	
H9A	0.4949	0.7541	0.1470	0.081*	
H9B	0.5817	0.6175	0.2403	0.081*	
C10	0.6327 (3)	0.7735 (2)	0.26206 (19)	0.0700 (6)	
H10A	0.6689	0.7291	0.3413	0.084*	
H10B	0.5820	0.8658	0.2480	0.084*	
N2	0.7482 (2)	0.78048 (16)	−0.04611 (14)	0.0591 (5)	
N3	0.7144 (2)	0.99200 (16)	−0.08345 (15)	0.0647 (5)	
C13	0.8010 (3)	0.6783 (3)	0.5629 (2)	0.0762 (7)	
C14	0.7534 (3)	0.8061 (2)	0.5600 (2)	0.0788 (7)	
H14A	0.8135	0.8558	0.5296	0.095*	
C15	0.6152 (3)	0.8589 (2)	0.6032 (2)	0.0691 (7)	
H15A	0.5821	0.9455	0.6030	0.083*	
C16	0.5225 (2)	0.78710 (18)	0.64746 (18)	0.0573 (6)	
C17	0.5794 (3)	0.65813 (19)	0.64870 (19)	0.0604 (6)	
C18	0.7182 (3)	0.6007 (2)	0.6075 (2)	0.0690 (6)	
H18A	0.7540	0.5134	0.6100	0.083*	
C19	0.3729 (3)	0.8500 (2)	0.69230 (19)	0.0678 (6)	
C20	0.2382 (2)	0.83360 (19)	0.65607 (17)	0.0582 (5)	
C21	0.1380 (3)	0.7875 (3)	0.7494 (2)	0.0928 (9)	
H21A	0.0900	0.7320	0.7342	0.111*	
H21B	0.1660	0.7684	0.8303	0.111*	
C22	0.0889 (3)	0.9228 (3)	0.6814 (2)	0.0884 (8)	
H22A	0.0864	0.9871	0.7204	0.106*	
H22B	0.0105	0.9507	0.6244	0.106*	
N6	0.2577 (2)	0.69776 (17)	0.41577 (15)	0.0633 (5)	
N5	0.26550 (18)	0.87964 (14)	0.44550 (14)	0.0533 (4)	
C12	0.7074 (2)	0.94269 (18)	0.02927 (17)	0.0536 (5)	
H12A	0.6907	0.9894	0.0846	0.064*	
C11	0.7406 (3)	0.8883 (2)	−0.12474 (18)	0.0662 (6)	
H11A	0.7525	0.8930	−0.2042	0.079*	
N1	0.72688 (16)	0.81780 (13)	0.05495 (12)	0.0423 (4)	
N4	0.25081 (17)	0.79612 (14)	0.54907 (13)	0.0464 (4)	
C24	0.2467 (2)	0.68990 (19)	0.52741 (19)	0.0588 (5)	
H24A	0.2371	0.6190	0.5847	0.071*	
C23	0.2694 (2)	0.81488 (19)	0.37037 (18)	0.0542 (5)	
H23A	0.2797	0.8479	0.2904	0.065*	
O1	0.85746 (19)	0.50900 (16)	0.25590 (17)	0.0984 (6)	
O2	0.3590 (2)	0.9191 (2)	0.75643 (18)	0.1132 (7)	

### Atomic displacement parameters (Å^2^)

**Table:**

	*U*^11^	*U*^22^	*U*^33^	*U*^12^	*U*^13^	*U*^23^
F1	0.0625 (9)	0.1236 (13)	0.1359 (14)	−0.0376 (9)	0.0205 (9)	−0.0597 (11)
F2	0.0660 (8)	0.0637 (8)	0.0980 (10)	−0.0180 (6)	−0.0012 (7)	−0.0458 (7)
F3	0.0818 (12)	0.1246 (14)	0.1586 (17)	−0.0256 (10)	−0.0010 (10)	−0.0595 (13)
F4	0.1023 (11)	0.0479 (7)	0.1065 (11)	−0.0320 (7)	−0.0159 (8)	−0.0061 (7)
C1	0.0459 (12)	0.0778 (16)	0.0745 (15)	−0.0183 (11)	−0.0014 (10)	−0.0297 (13)
C2	0.0566 (14)	0.0630 (14)	0.0824 (16)	−0.0086 (11)	−0.0069 (11)	−0.0345 (12)
C3	0.0601 (13)	0.0419 (11)	0.0718 (14)	−0.0118 (10)	−0.0159 (11)	−0.0146 (10)
C4	0.0504 (11)	0.0401 (10)	0.0495 (11)	−0.0122 (9)	−0.0116 (9)	−0.0065 (8)
C5	0.0519 (12)	0.0468 (11)	0.0566 (12)	−0.0101 (9)	−0.0101 (9)	−0.0215 (9)
C6	0.0542 (13)	0.0580 (13)	0.0741 (15)	−0.0230 (10)	−0.0108 (11)	−0.0225 (11)
C7	0.0643 (13)	0.0430 (12)	0.0546 (12)	−0.0207 (10)	−0.0066 (10)	−0.0001 (9)
C8	0.0522 (11)	0.0431 (11)	0.0444 (11)	−0.0203 (9)	−0.0006 (9)	−0.0075 (8)
C9	0.0594 (13)	0.0629 (14)	0.0797 (16)	−0.0295 (11)	0.0040 (11)	−0.0068 (12)
C10	0.0797 (16)	0.0751 (15)	0.0522 (13)	−0.0272 (13)	0.0119 (11)	−0.0155 (11)
N2	0.0828 (13)	0.0495 (10)	0.0472 (10)	−0.0189 (9)	−0.0041 (8)	−0.0197 (8)
N3	0.0933 (14)	0.0466 (10)	0.0496 (11)	−0.0238 (9)	−0.0031 (9)	−0.0048 (8)
C13	0.0669 (16)	0.0743 (17)	0.0896 (18)	−0.0180 (14)	−0.0208 (13)	−0.0258 (14)
C14	0.0871 (19)	0.0678 (16)	0.0901 (18)	−0.0367 (15)	−0.0249 (15)	−0.0121 (14)
C15	0.0893 (18)	0.0437 (12)	0.0798 (16)	−0.0221 (12)	−0.0336 (14)	−0.0140 (11)
C16	0.0774 (15)	0.0406 (11)	0.0577 (13)	−0.0164 (10)	−0.0262 (11)	−0.0146 (9)
C17	0.0800 (16)	0.0407 (11)	0.0635 (14)	−0.0227 (11)	−0.0236 (11)	−0.0075 (10)
C18	0.0771 (16)	0.0447 (12)	0.0835 (16)	−0.0082 (12)	−0.0308 (13)	−0.0198 (11)
C19	0.0935 (18)	0.0516 (13)	0.0608 (14)	−0.0151 (12)	−0.0176 (12)	−0.0253 (11)
C20	0.0743 (14)	0.0527 (12)	0.0469 (12)	−0.0143 (11)	−0.0016 (10)	−0.0210 (10)
C21	0.117 (2)	0.112 (2)	0.0538 (15)	−0.0430 (19)	0.0201 (14)	−0.0304 (15)
C22	0.0852 (18)	0.094 (2)	0.0825 (18)	−0.0074 (15)	0.0070 (14)	−0.0493 (16)
N6	0.0885 (13)	0.0544 (11)	0.0570 (11)	−0.0311 (10)	0.0008 (9)	−0.0215 (9)
N5	0.0702 (11)	0.0415 (9)	0.0490 (10)	−0.0234 (8)	0.0010 (8)	−0.0078 (8)
C12	0.0735 (14)	0.0383 (11)	0.0507 (12)	−0.0181 (10)	−0.0012 (10)	−0.0151 (9)
C11	0.0932 (17)	0.0598 (14)	0.0409 (12)	−0.0215 (12)	−0.0025 (11)	−0.0111 (11)
N1	0.0511 (9)	0.0361 (8)	0.0403 (8)	−0.0138 (7)	−0.0024 (7)	−0.0117 (7)
N4	0.0597 (10)	0.0391 (8)	0.0428 (9)	−0.0187 (7)	−0.0004 (7)	−0.0121 (7)
C24	0.0857 (15)	0.0456 (12)	0.0534 (13)	−0.0350 (11)	0.0014 (10)	−0.0105 (9)
C23	0.0663 (13)	0.0528 (12)	0.0447 (11)	−0.0221 (10)	0.0050 (9)	−0.0140 (9)
O1	0.0834 (12)	0.0565 (10)	0.1217 (15)	−0.0247 (9)	−0.0031 (10)	0.0301 (10)
O2	0.1266 (16)	0.1143 (15)	0.1246 (16)	−0.0233 (12)	−0.0190 (12)	−0.0879 (14)

### Geometric parameters (Å, °)

**Table:**

F1—C1	1.357 (3)	C13—C14	1.365 (3)
F2—C5	1.354 (2)	C14—C15	1.364 (3)
F3—C13	1.348 (3)	C14—H14A	0.9300
F4—C17	1.348 (2)	C15—C16	1.390 (3)
C1—C6	1.366 (3)	C15—H15A	0.9300
C1—C2	1.366 (3)	C16—C17	1.382 (3)
C2—C3	1.371 (3)	C16—C19	1.483 (3)
C2—H2A	0.9300	C17—C18	1.371 (3)
C3—C4	1.396 (3)	C18—H18A	0.9300
C3—H3A	0.9300	C19—O2	1.215 (2)
C4—C5	1.378 (3)	C19—C20	1.493 (3)
C4—C7	1.486 (3)	C20—N4	1.437 (2)
C5—C6	1.364 (3)	C20—C22	1.498 (3)
C6—H6A	0.9300	C20—C21	1.502 (3)
C7—O1	1.209 (2)	C21—C22	1.464 (4)
C7—C8	1.498 (3)	C21—H21A	0.9700
C8—N1	1.436 (2)	C21—H21B	0.9700
C8—C9	1.494 (3)	C22—H22A	0.9700
C8—C10	1.503 (3)	C22—H22B	0.9700
C9—C10	1.470 (3)	N6—C24	1.303 (3)
C9—H9A	0.9700	N6—C23	1.342 (3)
C9—H9B	0.9700	N5—C23	1.305 (2)
C10—H10A	0.9700	N5—N4	1.363 (2)
C10—H10B	0.9700	C12—N1	1.327 (2)
N2—C11	1.309 (3)	C12—H12A	0.9300
N2—N1	1.361 (2)	C11—H11A	0.9300
N3—C12	1.307 (2)	N4—C24	1.330 (2)
N3—C11	1.348 (3)	C24—H24A	0.9300
C13—C18	1.364 (3)	C23—H23A	0.9300
			
F1—C1—C6	117.4 (2)	C17—C16—C15	116.4 (2)
F1—C1—C2	118.9 (2)	C17—C16—C19	124.0 (2)
C6—C1—C2	123.7 (2)	C15—C16—C19	119.55 (19)
C1—C2—C3	117.9 (2)	F4—C17—C18	118.42 (19)
C1—C2—H2A	121.1	F4—C17—C16	117.9 (2)
C3—C2—H2A	121.1	C18—C17—C16	123.6 (2)
C2—C3—C4	121.47 (19)	C13—C18—C17	116.4 (2)
C2—C3—H3A	119.3	C13—C18—H18A	121.8
C4—C3—H3A	119.3	C17—C18—H18A	121.8
C5—C4—C3	116.77 (19)	O2—C19—C16	119.7 (2)
C5—C4—C7	124.50 (17)	O2—C19—C20	119.5 (2)
C3—C4—C7	118.72 (17)	C16—C19—C20	120.83 (17)
F2—C5—C6	117.88 (17)	N4—C20—C19	116.14 (18)
F2—C5—C4	118.42 (18)	N4—C20—C22	116.23 (18)
C6—C5—C4	123.66 (18)	C19—C20—C22	117.79 (19)
C5—C6—C1	116.47 (19)	N4—C20—C21	117.26 (19)
C5—C6—H6A	121.8	C19—C20—C21	118.64 (19)
C1—C6—H6A	121.8	C22—C20—C21	58.42 (17)
O1—C7—C4	120.29 (19)	C22—C21—C20	60.67 (17)
O1—C7—C8	119.54 (19)	C22—C21—H21A	117.7
C4—C7—C8	120.14 (16)	C20—C21—H21A	117.7
N1—C8—C9	116.98 (16)	C22—C21—H21B	117.7
N1—C8—C7	115.56 (15)	C20—C21—H21B	117.7
C9—C8—C7	118.05 (17)	H21A—C21—H21B	114.8
N1—C8—C10	117.75 (16)	C21—C22—C20	60.91 (16)
C9—C8—C10	58.74 (14)	C21—C22—H22A	117.7
C7—C8—C10	117.95 (17)	C20—C22—H22A	117.7
C10—C9—C8	60.96 (14)	C21—C22—H22B	117.7
C10—C9—H9A	117.7	C20—C22—H22B	117.7
C8—C9—H9A	117.7	H22A—C22—H22B	114.8
C10—C9—H9B	117.7	C24—N6—C23	102.17 (16)
C8—C9—H9B	117.7	C23—N5—N4	102.04 (15)
H9A—C9—H9B	114.8	N3—C12—N1	111.64 (17)
C9—C10—C8	60.30 (14)	N3—C12—H12A	124.2
C9—C10—H10A	117.7	N1—C12—H12A	124.2
C8—C10—H10A	117.7	N2—C11—N3	115.84 (18)
C9—C10—H10B	117.7	N2—C11—H11A	122.1
C8—C10—H10B	117.7	N3—C11—H11A	122.1
H10A—C10—H10B	114.9	C12—N1—N2	108.90 (15)
C11—N2—N1	101.85 (15)	C12—N1—C8	132.04 (15)
C12—N3—C11	101.76 (17)	N2—N1—C8	119.02 (14)
F3—C13—C18	118.3 (2)	C24—N4—N5	108.55 (15)
F3—C13—C14	118.2 (3)	C24—N4—C20	131.78 (17)
C18—C13—C14	123.4 (3)	N5—N4—C20	119.64 (15)
C15—C14—C13	118.1 (2)	N6—C24—N4	111.46 (18)
C15—C14—H14A	120.9	N6—C24—H24A	124.3
C13—C14—H14A	120.9	N4—C24—H24A	124.3
C14—C15—C16	122.0 (2)	N5—C23—N6	115.78 (18)
C14—C15—H15A	119.0	N5—C23—H23A	122.1
C16—C15—H15A	119.0	N6—C23—H23A	122.1
			
F1—C1—C2—C3	−178.8 (2)	C17—C16—C19—O2	132.8 (2)
C6—C1—C2—C3	1.6 (4)	C15—C16—C19—O2	−45.8 (3)
C1—C2—C3—C4	0.9 (3)	C17—C16—C19—C20	−49.5 (3)
C2—C3—C4—C5	−2.6 (3)	C15—C16—C19—C20	131.9 (2)
C2—C3—C4—C7	178.48 (19)	O2—C19—C20—N4	156.3 (2)
C3—C4—C5—F2	−175.89 (17)	C16—C19—C20—N4	−21.4 (3)
C7—C4—C5—F2	2.9 (3)	O2—C19—C20—C22	11.7 (3)
C3—C4—C5—C6	2.1 (3)	C16—C19—C20—C22	−166.0 (2)
C7—C4—C5—C6	−179.09 (19)	O2—C19—C20—C21	−55.6 (3)
F2—C5—C6—C1	178.18 (18)	C16—C19—C20—C21	126.7 (2)
C4—C5—C6—C1	0.2 (3)	N4—C20—C21—C22	−105.5 (2)
F1—C1—C6—C5	178.23 (19)	C19—C20—C21—C22	106.7 (2)
C2—C1—C6—C5	−2.1 (3)	N4—C20—C22—C21	107.2 (2)
C5—C4—C7—O1	−137.7 (2)	C19—C20—C22—C21	−108.2 (2)
C3—C4—C7—O1	41.0 (3)	C11—N3—C12—N1	−0.4 (2)
C5—C4—C7—C8	44.3 (3)	N1—N2—C11—N3	−0.8 (3)
C3—C4—C7—C8	−136.94 (19)	C12—N3—C11—N2	0.8 (3)
O1—C7—C8—N1	−152.1 (2)	N3—C12—N1—N2	−0.1 (2)
C4—C7—C8—N1	25.9 (2)	N3—C12—N1—C8	−177.66 (18)
O1—C7—C8—C9	−6.6 (3)	C11—N2—N1—C12	0.5 (2)
C4—C7—C8—C9	171.43 (17)	C11—N2—N1—C8	178.46 (17)
O1—C7—C8—C10	60.9 (3)	C9—C8—N1—C12	93.1 (2)
C4—C7—C8—C10	−121.1 (2)	C7—C8—N1—C12	−121.0 (2)
N1—C8—C9—C10	−107.59 (19)	C10—C8—N1—C12	26.0 (3)
C7—C8—C9—C10	107.3 (2)	C9—C8—N1—N2	−84.3 (2)
N1—C8—C10—C9	106.28 (19)	C7—C8—N1—N2	61.6 (2)
C7—C8—C10—C9	−107.5 (2)	C10—C8—N1—N2	−151.36 (17)
F3—C13—C14—C15	178.6 (2)	C23—N5—N4—C24	−0.2 (2)
C18—C13—C14—C15	−0.7 (4)	C23—N5—N4—C20	−178.35 (17)
C13—C14—C15—C16	−1.0 (3)	C19—C20—N4—C24	115.3 (2)
C14—C15—C16—C17	1.9 (3)	C22—C20—N4—C24	−99.5 (3)
C14—C15—C16—C19	−179.5 (2)	C21—C20—N4—C24	−33.2 (3)
C15—C16—C17—F4	176.35 (18)	C19—C20—N4—N5	−67.0 (2)
C19—C16—C17—F4	−2.2 (3)	C22—C20—N4—N5	78.1 (2)
C15—C16—C17—C18	−1.1 (3)	C21—C20—N4—N5	144.4 (2)
C19—C16—C17—C18	−179.7 (2)	C23—N6—C24—N4	0.2 (2)
F3—C13—C18—C17	−177.9 (2)	N5—N4—C24—N6	0.0 (2)
C14—C13—C18—C17	1.4 (3)	C20—N4—C24—N6	177.81 (19)
F4—C17—C18—C13	−177.89 (19)	N4—N5—C23—N6	0.4 (2)
C16—C17—C18—C13	−0.5 (3)	C24—N6—C23—N5	−0.4 (2)

### Hydrogen-bond geometry (Å, °)

**Table:**

*D*—H···*A*	*D*—H	H···*A*	*D*···*A*	*D*—H···*A*
C12—H12A···O2^i^	0.93	2.35	3.268 (2)	171.
C24—H24A···O1^ii^	0.93	2.37	3.265 (3)	161.

Symmetry codes: (i) −*x*+1, −*y*+2, −*z*+1; (ii) −*x*+1, −*y*+1, −*z*+1.

## References

[bb1] Che, X.-Y. & Zhang, W.-N. (2009). *Eur. J. Med. Chem.* **44**, 4218–4226. 10.1016/j.ejmech.2009.05.018 19539408

[bb2] Higashi, T. (1995). *ABSCOR* Rigaku Corporation, Tokyo, Japan.

[bb3] Lieven, M. & Leo, J. J. (2005). *J. Med. Chem.* **48**, 2184–2193.

[bb4] Molecular Structure Corporation (1992). *TEXSAN* MSC, The Woodlands, Texas, USA.

[bb5] Rigaku (1997). *R-AXIS II Software* Rigaku Corporation, Tokyo, Japan.

[bb6] Sheldrick, G. M. (2008). *Acta Cryst.* A**64**, 112–122. 10.1107/S0108767307043930 18156677

[bb7] Tarun, K. M., Debasis, D. & Chittaranjan, S. (1998). *Inorg. Chem.* **37**, 1672–1678.

